# Integrating Sensory Evaluation and Metabolomics to Reveal the Metabolic Basis of Taste and Flesh Color in Melon (*Cucumis melo* L.)

**DOI:** 10.3390/metabo16060368

**Published:** 2026-05-28

**Authors:** Yu Zhou, Binbin Li, Weizhong He, Fengjuan Liu, Yingying Fan, Jiangtao Du, Xing Cui, Weijia Lian, Qi Shen, Yan Wang, Zhongkai Zhao, Cheng Wang

**Affiliations:** 1 College of Smart Agriculture (Research Institute), Xinjiang University, Urumqi 830017, China; 2 Institute of Quality Standards & Testing Technology for Agro-Products, Academy of Agricultural Sciences of Xinjiang Uyghur Autonomous Region, Urumqi 830091, China

**Keywords:** *Cucumis melo*, sensory evaluation, untargeted metabolomics, taste, flesh color

## Abstract

**Background**: The sensory quality of melon (*Cucumis melo* L.) is determined by the complex interplay of metabolites within the fruit. However, the underlying metabolic mechanisms based on consumer sensory experience remain underexplored. **Methods**: Sensory evaluation was conducted on twelve melon cultivars, recording flesh color and quantitatively scoring acidity, sweetness, firmness, and aroma intensity. Based on the sensory results, eight cultivars were selected to establish two contrasting groups: sweet-type vs. acidic-type and orange-fleshed vs. green-fleshed. Untargeted metabolomics (UPLC-QTOF-MS) was then performed to analyze the samples, and differential metabolites were screened using OPLS-DA combined with univariate analysis. **Results**: Pathway enrichment analysis revealed that the key distinction between sweet and acidic taste profiles was associated with the specific accumulation of citric acid within the tricarboxylic acid (TCA) cycle in the acidic-type group. Regarding flesh color, the orange-fleshed group was enriched with carotenoid derivatives like β-citraurinene and the oxidized tocopherol product α-tocopherolquinone, whereas the green-fleshed group mainly accumulated phytol, a chlorophyll degradation product, along with more abundant terpenoids. **Conclusions**: By integrating sensory phenotyping with metabolomic analysis, this study identified key differential metabolites and candidate pathways associated with taste and color in melon, providing metabolic insights and data resources for quality evaluation and regulation.

## 1. Introduction

Melon (*Cucumis melo* L.) belongs to the genus *Cucumis*, tribe Cucurbiteae Ser., and family Cucurbitaceae Juss [1]. It is cultivated in regions ranging from temperate to tropical zones and is widely popular among consumers for its distinctive flavor and rich nutrition [2]. The sensory quality and nutritional basis of melon fruit are collectively shaped by various components, including soluble sugars, organic acids, carotenoids, vitamin C, and volatile aroma compounds [3,4]. Among these, sweetness, sourness, and flesh color are key sensory attributes influencing consumer preference [5]. Therefore, systematically elucidating the metabolite differences underlying distinct sensory experiences is of great significance for understanding the mechanisms of melon quality formation, addressing the challenge of varietal homogenization, and developing diversified quality traits.

Systematic sensory evaluation of fruit serves as a crucial starting point for linking complex phenotypes to their intrinsic material basis [6,7]. It translates intuitive attributes perceptible by consumers into quantifiable data, thereby providing a basis for the precise screening of experimental materials [8]. For instance, sensory analysis of peach fruit first established the association between red flesh and diminished flavor, which subsequently guided studies revealing its metabolic and genetic basis [9]. A study on pears found that integrating sensory evaluation with metabolite analysis identified key metabolites driving consumer preference [10]. In melon, similar research strategies have also been applied. For example, Escribano et al. [11] established a comprehensive sensory characterization protocol for melon. Menezes Ayres et al. [12] conducted descriptive analysis and consumer tests on 15 cantaloupe varieties, identifying sweet and oversweet flavor, as well as intense orange and richness of color, as key attributes driving consumer preference. Kaleem et al. [13] quantified melon flavor perception through sensory evaluation and integrated untargeted metabolomics to dissect its metabolic differences. These studies demonstrate that using sensory evaluation as a screening tool can effectively identify contrasting materials with target phenotypes, thereby providing a clear research premise for downstream analyses such as metabolomics.

Untargeted metabolomics is a high-throughput and unbiased method for detecting all small-molecule metabolites within a biological sample [14]. In fruit quality research, this technique has been widely applied to identify flavor compounds and elucidate metabolic pathways in fruits such as strawberry [15], apple [16], and grape [17]. In melon, studies have found that sucrose gradually becomes the dominant sugar during late ripening due to increased activities of sucrose phosphate synthase and sucrose phosphate phosphatase [18]. Metabolomic analysis of two melon cultivars, ‘Tianbao’ and ‘Xiaocuigua’, revealed that citric acid and malic acid are the predominant organic acids, exhibiting distinct accumulation patterns during fruit development [19]. Carotenoids are key factors affecting melon flesh color [20]. However, studies using untargeted metabolomics to dissect carotenoid metabolism in melon remain limited. In fact, untargeted approaches have been successfully used to identify carotenoid derivatives, including eight carotenoids, in goji berry (*Lycium barbarum* L.), providing an important reference for their application in melon carotenoid research [21]. Beyond these studies focusing on specific metabolites, large-scale untargeted metabolomics analyses have also been employed to reveal overall metabolic differences among melon groups. For example, Moing et al. [22] used six complementary untargeted metabolomic platforms to systematically analyze 44 melon accessions, uncovering metabolic differences across various melon groups. Collectively, these studies have laid a foundation for our understanding of the molecular mechanisms underlying quality traits such as sugars, acids, and pigments in melon.

In melon research, studies that employ standardized sensory evaluation for targeted sample grouping to investigate the metabolic basis of taste and color remain limited. This study integrates sensory evaluation with untargeted metabolomics to reveal the metabolite foundations underlying sweetness vs. sourness and orange vs. green flesh color in melon. We first conducted a standardized sensory evaluation on twelve melon cultivars. Based on these results, we selected two groups of cultivars that exhibited opposing profiles in sweetness and sourness while maintaining consistency in other sensory attributes, establishing a sweet-type (ST) group and an acidic-type (AT) group. Concurrently, we selected cultivars with typical orange and green flesh colors to establish an orange-fleshed (OF) group and a green-fleshed (GF) group. Subsequently, untargeted metabolomic analysis was performed based on these groupings to screen for differentially accumulated metabolites (DAMs) and explore the associated metabolic pathways. The untargeted approach enables a systemic perspective beyond isolated analyses of individual components, facilitating the elucidation of metabolic networks underlying sensory quality. This study aims to provide new insights into the material basis and metabolic pathways governing key sensory traits in melon, and to offer a metabolic-level reference for quality evaluation and utilization.

## 2. Materials and Methods

### 2.1. Fruit Materials

Twelve melon cultivars used in this study, namely Baihe (BH), Fengwei 4 (FW4), Fengwei 8 (FW8), Huangzuixian (HZX), Jan-86, Jinyingyindi (JYYD), Junxiu (JX), K11-39, K11-40, Lvbaoshi 2 (LBS2), Qinghuami (QHM), and Xinxuelihong (XXLH), were collected by our laboratory and planted at the Comprehensive Experimental Station (Anningqu) of the Xinjiang Academy of Agricultural Sciences. These cultivars include both commonly available commercial melon types in China and some germplasm resources intended for commercial cultivar development, covering white, green, and orange flesh colors. Fruits with uniform maturity, consistent shape, and no signs of pests, diseases, or mechanical damage were selected for the study. For each cultivar, three biological replicates were collected. Following harvest, the fruits were immediately transported to the laboratory in insulated boxes with ice packs.

### 2.2. Sensory Evaluation

The sensory evaluation panel consisted of eight trained students majoring in food science. All panelists were screened to confirm the absence of olfactory, gustatory, or visual (color vision) impairments, and none reported aversion or allergic reactions to melon. For the four attributes of acidity, sweetness, firmness, and aroma intensity, the panelists were trained both collectively and individually based on the reference substance setup method described by Sandra Escribano et al. [11]. Training sessions were held twice a week, one hour per session, for a total of four weeks, until the panelists reached acceptable levels in discriminative ability, result reproducibility, and consistency in concept understanding. Fresh melon flesh samples from the twelve cultivars were served at room temperature in a completely randomized order. Panelists rated the four key attributes using a 0–10 point scale with integer values, where 0 represented extremely weak, 5 represented moderate, and 10 represented extremely intense. In addition, flesh color was visually assessed by the panelists and classified into white, green, and orange categories; all samples were accompanied by cross-sectional images as a reference for classification. After evaluating each sample, panelists rinsed their mouths with water and waited at least 60 s to alleviate sensory fatigue. The results were expressed as mean ± SD. Intraclass correlation coefficient (ICC, two-way random model, absolute agreement) was used to assess the consistency of panelists’ ratings, with an ICC value of >0.75 indicating good consistency.

### 2.3. Sample Preparation and Metabolite Extraction

Samples were uniformly collected from three equidistant points around the fruit equatorial region and immediately placed into 50 mL centrifuge tubes. The tubes were flash-frozen in liquid nitrogen and then stored at −80 °C until further processing. Prior to analysis, the frozen samples were ground into a fine powder using a high-throughput vibration ball mill (Ant Source Scientific Instrument Co., Ltd., Beijing, China) at 27 Hz for 2 min. Exactly 0.100 g of the powder was accurately weighed into a 1.5 mL centrifuge tube, followed by the addition of 300 μL of extraction solvent. The extraction solvent was an aqueous solution containing 0.125% (*v*/*v*) formic acid (CAS#64-18-6; Thermo Fisher Scientific (China) Co., Ltd., Shanghai, China) and 50% (*v*/*v*) methanol (CAS#67-56-1; Thermo Fisher Scientific (China) Co., Ltd., Shanghai, China). The mixture was vortexed for 10 s, sonicated in a water bath at 20 °C for 15 min, and then centrifuged at 13,000 rpm for 15 min. The resulting supernatant was filtered through a 0.22 μm hydrophilic syringe filter to obtain the final sample solution for instrumental analysis.

### 2.4. Untargeted Metabolomic Analysis of Melon Fruits

Separation and detection of the samples were performed using an ultra-performance liquid chromatography–quadrupole time-of-flight mass spectrometry (UPLC-QTOF-MS) system (UPLC: Waters Corporation, Milford, MA, USA; MS: AB Sciex Pte. Ltd., Singapore).

Chromatographic separation was achieved on a Waters ACQUITY UPLC HSS T3 column (1.8 µm, 2.1 mm × 100 mm; Waters Corporation, Milford, MA, USA). The mobile phases consisted of A, 0.2% formic acid in water, and B, acetonitrile (CAS#75-05-8; Thermo Fisher Scientific (China) Co., Ltd., Shanghai, China). The column temperature was maintained at 40 °C, the autosampler temperature was set at 4 °C, and the flow rate was 0.300 mL/min. The injection volume was 2 µL.

Mass spectrometric analysis was conducted on a SCIEX ZenoTOF^TM^ 7600 system (SCIEX, Framingham, MA, USA). Metabolomic data from melon samples were acquired in information-dependent acquisition (IDA) mode, which provided both TOF-MS primary (MS1) scans and high-sensitivity product ion (MS2) scans for each sample. The IDA cycle (551 ms) comprised a TOF-MS scan (accumulation time: 50 ms) and a product ion scan in high-sensitivity mode (accumulation time: 30 ms), with dynamic background subtraction enabled. The *m*/*z* range for MS1 scanning was 100–1000 Da, and that for MS2 scanning was 50–1000 Da. Throughout the sequence, quality control (QC) samples were injected every four sample runs to monitor instrument stability. Additionally, a calibration solution was delivered via CDS and introduced into the ion source through an APCI probe every ten sample injections for system calibration.

Raw high-resolution mass spectral data files (.wiff/.wiff2 format) were automatically generated and saved by the instrument control software (SCIEX OS V3.3.1.43). These files were subsequently imported into Progenesis QI V3.0 software (Waters Corporation, Milford, MA, USA) for peak extraction and retention time alignment. Metabolite identification was performed by matching the processed mass spectral data against the HMDB, MoNA, and METLIN™ MS/MS Library 2019 databases. The most appropriate metabolite for each feature was selected based on MS/MS fragmentation analysis, retention time matching, and match scores. According to the Metabolomics Standards Initiative (MSI), the identified metabolites in this study were assigned to confidence level 2 [23]. For spectral matching, the mass error tolerances for MS1 and MS2 were set at 10 ppm, and the retention time (Rt) error tolerance was set at 0.1 min. Subsequently, features with a relative standard deviation (RSD) greater than 30% and a detection rate below 80% in the QC samples were filtered out based on raw peak area. For zero or missing values in the filtered dataset, imputation was performed using one-fifth of the minimum positive value per row. Prior to statistical analysis, the data were subjected to unit variance (UV) scaling. Further data processing was performed as required during statistical analysis.

### 2.5. Determination of Organic Acids and Soluble Sugars by HPLC

An accurately weighed 0.500 g portion of melon powder was mixed with deionized water in a 10 mL volumetric flask. The mixture was extracted in a constant-temperature water bath at 45 °C for 1 h, with shaking every 10 min. After cooling, the volume was adjusted to 10 mL with deionized water and mixed thoroughly. The mixture was then centrifuged at 10,000 r/min for 10 min, and the supernatant was filtered through a 0.45 μm membrane. The resulting filtrate was used for the determination of soluble sugars and organic acids.

The analysis was performed on a Waters E2695 high-performance liquid chromatography system (Waters Corporation, Milford, MA, USA). Organic acids were separated on a Waters XBridge C18 column (5 μm, 4.6 mm × 250 mm; Waters Corporation, Milford, MA, USA) and detected by a UV detector at 210 nm. The column temperature was maintained at 40 °C, and the mobile phase was acetonitrile-water (78:22, *v*/*v*) at a flow rate of 1.0 mL/min. Soluble sugars were separated on a Macherey-Nagel Nucleodur 100-5 NH2-RP column (5 μm, 4.6 mm × 250 mm; Macherey-Nagel GmbH & Co. KG, Düren, Germany) and detected by a refractive index detector (RID). The column temperature was 40 °C, and the mobile phase was methanol-0.1% phosphoric acid (CAS#7664-38-2; Tianjin Fuyu Fine Chemical Co., Ltd., Tianjin, China) in water (2.5:97.5, *v*/*v*) at a flow rate of 1.0 mL/min. Quantification was performed using the external standard method. Reference standards included citric acid (CAS#77-92-9), malic acid (CAS#6915-15-7), sucrose (CAS#57-50-1), fructose (CAS#57-48-7) and glucose (CAS#492-62-6), all from ANPEL-TRACE Standard Technical Services (Shanghai) Co., Ltd. (Shanghai, China). Organic acid contents were expressed as g/kg fresh weight (FW), and soluble sugar contents as g/100 g FW.

### 2.6. Statistical Analysis

Organic acid and soluble sugar contents were analyzed by one-way analysis of variance (ANOVA), followed by Tukey’s HSD post hoc test for multiple comparisons. Multivariate statistical analysis of metabolomics data was performed on the Metware Cloud platform (https://cloud.metware.cn, accessed on 26 November 2025). The data were subjected to log2 transformation and UV scaling prior to principal component analysis (PCA). An orthogonal partial least squares-discriminant analysis (OPLS-DA) model was built based on the same preprocessed data, incorporating all features that passed QC filtering. During model construction, leave-one-out cross-validation (LOOCV) was applied, with one predictive component and two orthogonal components automatically determined by the platform. The model validity was assessed using 200 permutation tests. To further validate the robustness of the model, supplementary analysis was performed on the Workflow4Metabolomers platform (https://workflow4metabolomics.org, accessed on 1 March 2026) using 10-fold cross-validation and 100 permutation tests, and the classification accuracy, receiver operating characteristic (ROC) curve, and confusion matrix were calculated. DAMs were screened by integrating criteria from the OPLS-DA model, fold change (FC), and statistical significance. The specific thresholds were: variable importance in projection (VIP) > 1.0, FC > 1.5 (or <0.667), and a *p*-value < 0.05 after Welch’s *t*-test with false discovery rate (FDR) correction. Visualization plots, including volcano plots and hierarchical clustering heatmaps of DAMs, were generated by the Metware Cloud platform. Pathway enrichment analysis of DAMs was conducted on the MetaboAnalyst 6.0 platform (https://www.metaboanalyst.ca, accessed on 2 December 2025) using the hypergeometric test. KEGG pathways with an FDR-corrected *p*-value < 0.05 were considered significantly enriched. Data were preprocessed using Microsoft Excel 2021.

## 3. Results

### 3.1. Establishment of Contrasting Groups Based on Sensory Evaluation

**Figure 1:**
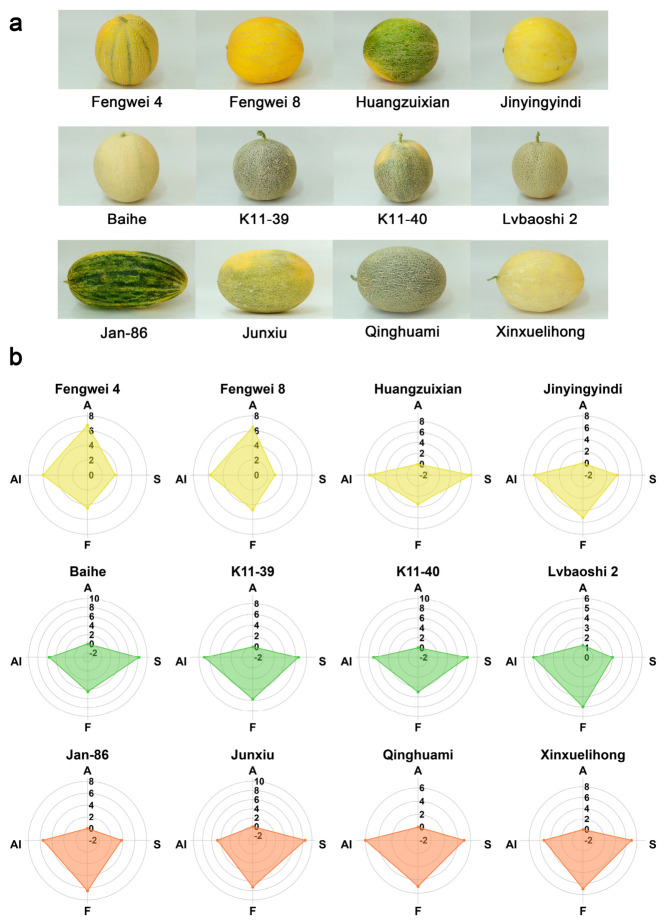
The twelve melon cultivars (**a**) and their sensory evaluation results presented as a radar chart (**b**). A—Acidity; S—Sweetness; F—Firmness; AI—Aroma Intensity.

This study first conducted a systematic sensory evaluation of twelve commercial melon cultivars (Figure 1a), focusing on four key attributes: acidity, sweetness, firmness, and aroma intensity. ICC analysis revealed that the ICC values for the four attributes were 0.997, 0.970, 0.863, and 0.763, respectively, indicating reliable consistency among panelists (Table S1). A radar chart of the sensory scores was constructed to visualize the results (Figure 1b), which clearly revealed significant sensory differences among the cultivars. Based on flesh color, the cultivars were categorized into three groups: Fengwei 4, Fengwei 8, Huangzuixian, and Jinyingyindi were classified as white-fleshed; Baihe, K11-39, K11-40, and Lvbaoshi 2 as green-fleshed; and Jan-86, Junxiu, Qinghuami, and Xinxuelihong as orange-fleshed (Figure S1). Regarding taste profiles, Fengwei 4 and Fengwei 8 exhibited pronounced sourness, while cultivars such as Baihe, Huangzuixian, Junxiu, and K11-40 scored notably high in sweetness.

To investigate the metabolic basis of these key sensory traits, two pairs of contrast groups were established based on the sensory scores, while minimizing differences in other sensory attributes. For the sweetness and sourness dimension, K11-40 and Huangzuixian were selected to form the ST group, whereas Fengwei 4 and Fengwei 8 constituted the AT group. For the flesh color dimension, Junxiu and Xinxuelihong were selected as the OF group, and Baihe and K11-39 as the GF group. This grouping strategy was designed to focus on the target traits, thereby establishing a clear phenotypic foundation for the subsequent comparative metabolomic analysis.

### 3.2. Multivariate Statistical Analysis of Metabolic Profiles

**Figure 2:**
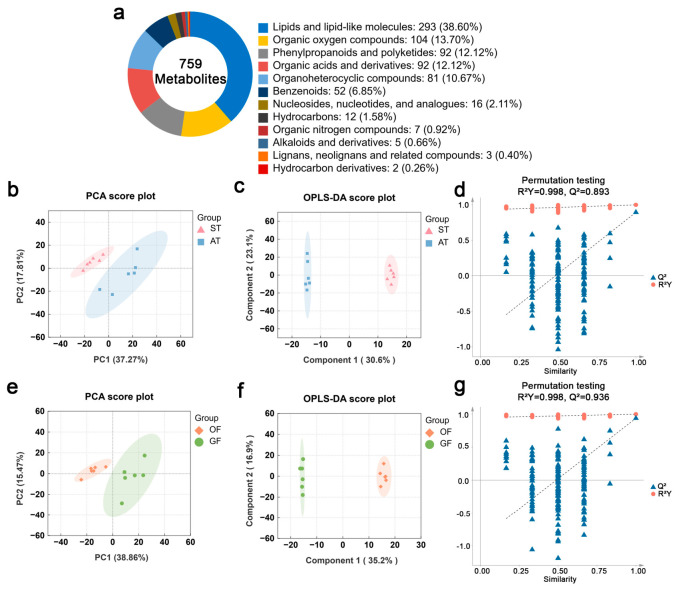
Global analysis of metabolic profiles and construction of discriminant models. (**a**) Pie chart of metabolite classification. (**b**) PCA score plot for the ST vs. AT comparison. (**c**) OPLS-DA score plot for the ST vs. AT comparison. (**d**) Permutation test result for the ST vs. AT OPLS-DA model. (**e**) PCA score plot for the OF vs. GF comparison. (**f**) OPLS-DA score plot for the OF vs. GF comparison. (**g**) Permutation test result for the OF vs. GF OPLS-DA model.

An untargeted metabolomic analysis was performed on the 24 samples from the eight selected melon groups using UPLC-QTOF-MS. A total of 759 metabolites were identified, comprising 523 in positive ion mode and 236 in negative ion mode (Table S2). These metabolites were categorized into 12 classes (Figure 2a): 293 lipids and lipid-like molecules; 104 organic oxygen compounds; 92 phenylpropanoids and polyketides; 92 organic acids and derivatives; 81 organoheterocyclic compounds; 52 benzenoids; 16 nucleosides, nucleotides, and analogs; 12 hydrocarbons; 7 organic nitrogen compounds; 5 alkaloids and derivatives; 3 lignans, neolignans, and related compounds; and 2 hydrocarbon derivatives.

To explore overall metabolic differences, PCA was first conducted. As shown in Figure 2b,e, for the ST vs. AT comparison, the first two principal components (PC1 and PC2) explained 37.27% and 17.81% of the total variance, respectively, with a cumulative explanation rate of 55.08%, indicating a systematic metabolic divergence. Similarly, clear separation was observed for the OF vs. GF comparison, with PC1 and PC2 explaining 38.86% and 15.47% of the variance, respectively, representing a cumulative 54.33%. These results preliminarily validated that sensory-based grouping reflected distinct metabolomic foundations. To enhance discrimination and identify key variables, supervised OPLS-DA was employed. Unlike PCA, which describes total variance, OPLS-DA filters out within-group variation unrelated to classification, thereby focusing on between-group differences. As shown in Figure 2c,f, the OPLS-DA score plots for both comparisons showed tight within-group clustering and clear between-group separation. Specifically, the model validation parameters for the two groups were R^2^Y = 0.998, Q^2^ = 0.893 (ST vs. AT) and R^2^Y = 0.998, Q^2^ = 0.936 (OF vs. GF), respectively, with Q^2^ values above 0.5 indicating good predictive ability (Figure 2d,g). Additionally, permutation tests yielded *p* < 0.005, and the intercept of the Q^2^ regression line was negative, confirming that the models were robust and free from overfitting (Figure S2). Furthermore, the classification accuracy, ROC curve, and confusion matrix from 10-fold cross-validation supported the good predictive performance of the models (Figure S3, Tables S3 and S4).

### 3.3. Screening of DAMs

**Figure 3:**
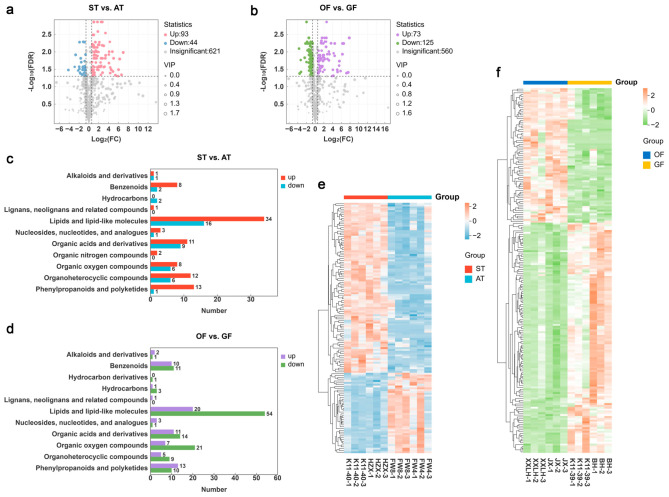
Analysis of DAMs. (**a**) Volcano plot of DAMs for the ST vs. AT comparison. (**b**) Volcano plot of DAMs for the OF vs. GF comparison. (**c**) Bar chart of DAMs classification for the ST vs. AT comparison. (**d**) Bar chart of DAMs classification for the OF vs. GF comparison. (**e**) Heatmap of DAMs for the ST vs. AT comparison. (**f**) Heatmap of DAMs for the OF vs. GF comparison.

Figure 3 presents the DAMs between the comparative groups. In the ST vs. AT comparison, volcano plots showed that a total of 137 significant DAMs were identified, with 93 up-regulated in the ST group and 44 up-regulated in the AT group (Figure 3a). Similarly, for the OF vs. GF comparison, 198 DAMs were screened, of which 73 were up-regulated and 125 were down-regulated (Figure 3b, Tables S5 and S6).

Hierarchical clustering analysis further revealed coordinated expression patterns of these DAMs. In the ST vs. AT comparison, the metabolites were naturally clustered into two main groups, which strictly corresponded to the high-abundance states in the ST and AT groups, respectively (Figure 3e). Similarly, the color comparison analysis also showed a clear metabolite clustering pattern, indicating a distinct correspondence between metabolite abundance profiles and flesh color phenotypes (Figure 3f).

The distribution of DAMs across chemical classes is shown in Figure 3c,d. In the ST vs. AT comparison, lipids and lipid-like molecules were the most abundant category, comprising 50 metabolites, of which 34 were up-regulated and 16 were down-regulated in the ST group. The ST group also showed up-regulation of 13 phenylpropanoids and polyketides and 12 organoheterocyclic compounds. In the OF vs. GF comparison, lipids and lipid-like molecules were again the major differential class, consisting of 74 metabolites: 20 were up-regulated in the OF group, and 54 were down-regulated.

### 3.4. KEGG Pathway Enrichment Analysis of DAMs

**Figure 4:**
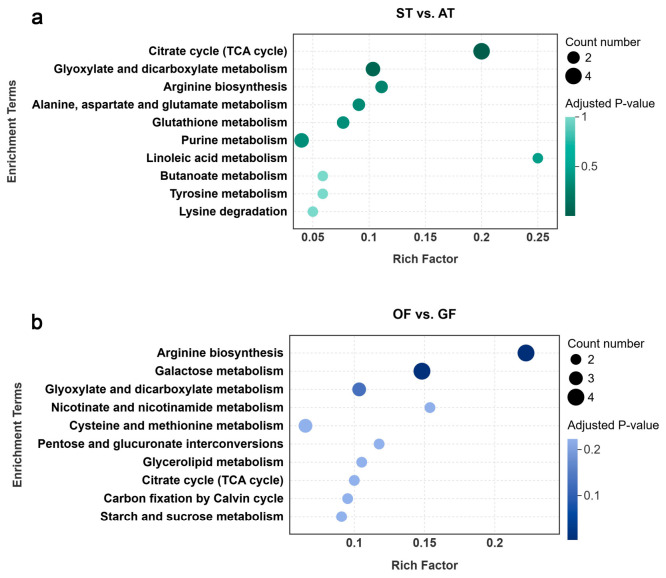
Bubble plots of the top 10 KEGG pathways enriched by DAMs. (**a**) ST vs. AT comparison. (**b**) OF vs. GF comparison.

To elucidate the core metabolic pathways involving the DAMs and to uncover potential metabolic regulatory mechanisms underlying the phenotypic differences, all DAMs from both contrast groups were subjected to KEGG pathway enrichment analysis. Figure 4 displays the top 10 enriched pathways for each group. It should be noted that some identified metabolites could not be mapped to standard KEGG pathways due to the current incompleteness of plant secondary metabolite databases.

For the ST vs. AT comparison, the top 10 enriched pathways included the citrate cycle (TCA cycle, 4/20, differentially accumulated metabolites/total metabolites in the pathway), glyoxylate and dicarboxylate metabolism (3/29), arginine biosynthesis (2/18), alanine, aspartate and glutamate metabolism (2/22), and glutathione metabolism (2/26). Among these, the TCA cycle exhibited a relatively higher rich factor and enrichment significance (Figure 4a). For the OF vs. GF comparison, the top 10 enriched pathways encompassed arginine biosynthesis (4/18), galactose metabolism (4/27), glyoxylate and dicarboxylate metabolism (3/29) and nicotinate and nicotinamide metabolism (2/13). In this group, the arginine biosynthesis pathway showed a rich factor greater than 0.2 and high enrichment significance (Figure 4b). Comparative analysis of the top 10 enriched pathways between the two contrast groups revealed that three pathways, namely the citrate cycle (TCA cycle), glyoxylate and dicarboxylate metabolism, and arginine biosynthesis, were common to both, suggesting potential pathway correlations and shared regulatory mechanisms underlying the metabolic regulation of different melon phenotypes (Tables S7 and S8).

### 3.5. Analysis of Key Metabolic Pathways

Based on the preliminary pathway enrichment results and considering pathway biological functions and fruit phenotypic traits, key metabolic pathways with potential biological relevance for fruit taste and color formation were selected for focused visualization and in-depth analysis.

**Figure 5:**
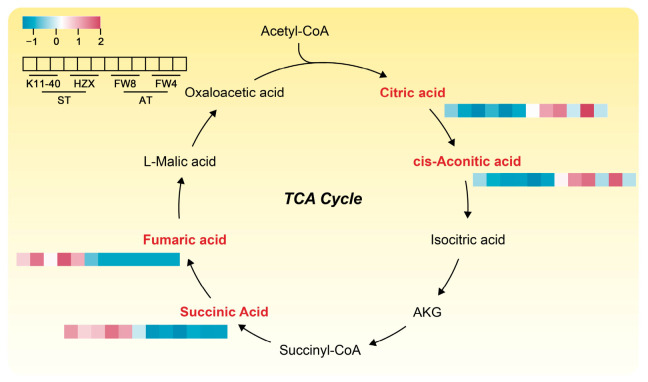
TCA cycle for the ST Group (K11-40 and HZX) and the AT Group (FW8 and FW4). DAMs are labeled in red. Heatmap colors were generated from standardized peak areas obtained by untargeted metabolomics, with red indicating up-regulation and blue indicating down-regulation.

In the ST vs. AT comparison, the TCA cycle was identified as the most significantly enriched pathway. The accumulation patterns of key intermediates in this pathway showed clear between-group differences (Figure 5). Citric acid and cis-aconitic acid (Rt = 1.19 min, [M+H]^+^ *m*/*z* 175.0233, Δ = −2.63 ppm) were significantly higher in the AT group fruits, with abundance levels 1.74 and 1.86 times those in the ST group, respectively. Conversely, both succinic acid and fumaric acid exhibited higher accumulation levels in the ST group fruits, at levels 8.04 and 23.05 times those in the AT group, respectively. These findings suggest a possible divergence in metabolic flux at the citrate node between the two fruit types. The substantial accumulation of citric acid in the AT group may be related to its stronger synthesis or relatively restricted downstream conversion, whereas in the ST group, more efficient conversion of citric acid toward downstream organic acids appeared to occur, potentially resulting in increased activity in the latter half of the TCA cycle. Therefore, the metabolic divergence at the citrate node may represent a key feature distinguishing the metabolomic profiles of sweet- and acidic-taste melons.

**Figure 6:**
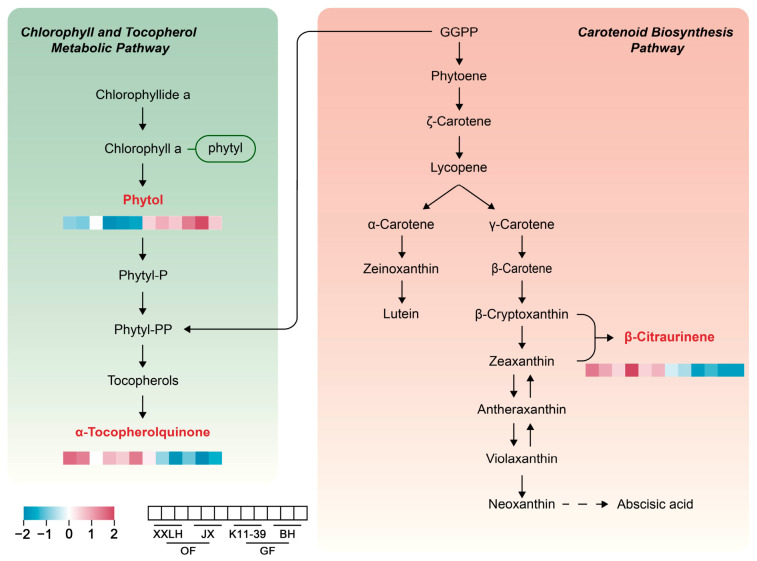
Metabolic pathways of chlorophyll and tocopherol metabolism and carotenoid biosynthesis for the OF Group (XXLH and JX) and the GF Group (K11-39 and BH). DAMs are labeled in red. Heatmap colors were generated from standardized peak areas obtained by untargeted metabolomics, with red indicating up-regulation and blue indicating down-regulation.

To investigate the metabolic basis of flesh color formation, key substances directly associated with pigment metabolism were selected from the DAMs, and a corresponding metabolic pathway map was constructed (Figure 6). The results indicated that phytol, involved in the chlorophyll metabolism pathway, was more abundant in the GF group fruits, with levels approximately 1.63 times those in the OF group. Concurrently, α-tocopherolquinone (Rt = 13.44 min, [M+Na]^+^ *m*/*z* 469.3626, Δ = −5.97 ppm), an oxidized product derived from the chlorophyll-tocopherol metabolism branch, and the carotenoid derivative β-citraurinene (Rt = 13.90 min, [M+Na]^+^ *m*/*z* 441.3166, Δ = 9.20 ppm) accumulated significantly in the OF group, with levels 2.78 and 5.73 times those in the GF group, respectively. Together, these differential metabolic profiles suggest that the GF group may maintain more active chlorophyll-related metabolism, with tocopherol metabolism favoring a non-oxidized, reduced state. In contrast, the OF group exhibited a metabolic shift toward the accumulation of oxidized tocopherol products and enhanced carotenoid accumulation, providing new clues for understanding the metabolic basis of melon flesh color formation.

### 3.6. HPLC Quantification of Organic Acids and Soluble Sugars

To validate the differences in sugars and organic acids observed in the untargeted metabolomics analysis, four melon cultivars, K11-40, HZX, FW8, and FW4, were selected from the ST and AT groups, with three biological replicates per cultivar. The contents of citric acid, malic acid, sucrose, fructose and glucose were determined by HPLC.

**Figure 7:**
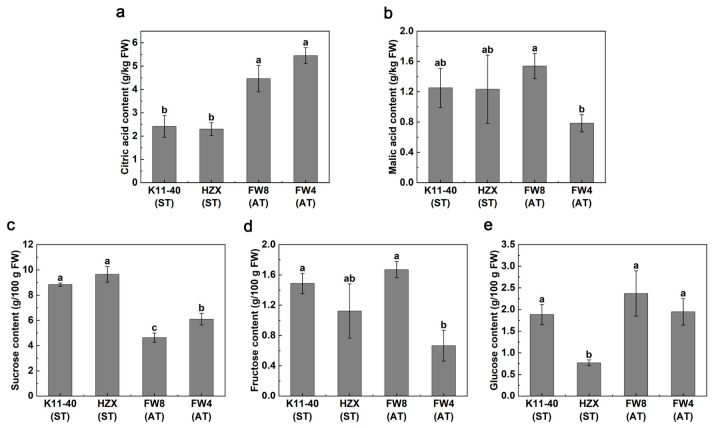
Contents of citric acid (**a**), malic acid (**b**), sucrose (**c**), fructose (**d**), and glucose (**e**) in the ST Group (K11-40 and HZX) and the AT Group (FW8 and FW4). Data are presented as mean ± SD from three biological replicates. Different letters indicate statistically significant differences determined by one-way ANOVA followed by Tukey’s HSD test (*p* < 0.05).

The quantitative results are shown in Figure 7. Citric acid levels were significantly higher in the AT group than in the ST group, with levels of 4.46 and 5.45 g/kg FW in AT cultivars FW8 and FW4, respectively, compared to 2.42 and 2.30 g/kg FW in ST cultivars K11-40 and HZX (Figure 7a). Malic acid showed no consistent difference between groups, except that its content in FW4 (0.78 g/kg FW) was significantly lower than in the other cultivars (Figure 7b). Nevertheless, citric acid levels were higher than malic acid levels in all cultivars, suggesting that the sourness of the fruit may be primarily contributed by citric acid.

Regarding soluble sugars, the sucrose contents in the ST cultivars K11-40 and HZX were as high as 8.83 and 9.64 g/100 g FW, respectively, significantly higher than those in the AT cultivars FW8 and FW4 at 4.63 and 6.10 g/100 g FW (Figure 7c). In contrast, fructose and glucose did not exhibit consistent inter-group differences, except that fructose in FW4 and glucose in HZX were significantly lower than in the other cultivars (Figure 7d,e).

Both untargeted metabolomics and HPLC analyses revealed significant accumulation of citric acid in the AT group, which further supports the important role of the TCA cycle in determining melon sourness.

## 4. Discussion

Fruit flavor, appearance, and nutritional quality are core factors influencing consumer choice [24]. For melon, sensory attributes such as acidity, sweetness, firmness, aroma intensity, and flesh color directly determine its market acceptance [12]. Human perception of fruit flavor involves the integration of multiple sensory systems. Traditional sensory evaluation serves as a scientific method to quantify such complex, subjective qualities. Its strength lies in its ability to directly and comprehensively reflect actual consumer perception, translating holistic experiences that are difficult to measure instrumentally into analyzable data [25,26]. In this study, based on sensory evaluation results, cultivars that exhibited opposing profiles in sweetness and sourness while maintaining consistency in other sensory attributes were selected to establish the ST and AT groups. Concurrently, cultivars with distinct flesh colors were selected to establish the OF and GF groups. Both grouping strategies were grounded in direct sensory judgment, providing clear targets for subsequent metabolomic analysis and enabling a focused search for metabolites associated with taste and color, respectively. The daily selection and consumption of fruit by consumers is essentially an integrated assessment of these sensory attributes. Further untargeted metabolomic analysis was conducted to identify metabolites associated with taste and color differences, thereby capturing changes in pathway activity and metabolic flux, and to explain the formation mechanisms of sensory perception from a metabolic perspective.

The composition and ratio of sugars and organic acids are central determinants of fruit taste and flavor balance [27]. In melon, citric acid and malic acid are the primary organic acids, with citric acid typically being predominant [28]. The accumulation of fruit acidity results from a dynamic balance between acid synthesis and degradation [29]. KEGG pathway enrichment analysis of DAMs in the ST vs. AT comparison revealed the TCA cycle as the most significantly enriched pathway. Within this pathway, citric acid accumulated to significantly higher levels in the AT group, suggesting a potential association with its perceived sourness. This finding aligns with conclusions from Cheng et al. [19] in oriental melons, who also identified citric acid as the key organic acid determining fruit acidity, with its variation being crucial for taste formation. Similarly, Umer et al. [30] found that organic acid levels in sour watermelon ‘SrW’ were significantly higher than those in sweet watermelon ‘203Z’, and that the expression of malate and citrate transporter genes was significantly correlated with sugar and organic acid contents, further supporting the key role of TCA cycle-derived organic acids in fruit flavor formation. Consistent with these observations, our HPLC quantification indicated that the significant accumulation of citric acid in the AT group followed the same trend as the untargeted metabolomics results. Notably, the levels of cis-aconitic acid were also higher in the AT group. Cis-aconitic acid is a key intermediate in the conversion of citric acid to isocitric acid, catalyzed by aconitase. The coordinated accumulation of citric acid and cis-aconitic acid led us to speculate that this metabolic step is relatively constrained in the AT group fruits, leading to insufficient degradation of citric acid. This metabolic bottleneck may be related to the regulation of key enzyme activities such as aconitase. For example, in jujube fruit, mutations in the promoter of the aconitase gene *ZjACO3* modulate its expression level, thereby determining the extent of citric acid accumulation [31]. This provides a potential molecular mechanism clue for understanding acidity differences among melon varieties. Meanwhile, HPLC quantification showed that absolute sucrose levels were significantly higher in the ST group. However, the difference in the untargeted metabolomics analysis did not reach the preset screening thresholds (FC > 1.5, FDR < 0.05), with actual values of FC = 1.358 and FDR = 0.058. Consequently, sucrose was not identified as a differential metabolite. This further highlights the potential key role of citric acid accumulation in distinguishing the taste profiles of the two groups. In contrast, succinic acid and fumaric acid were present at higher levels in the ST group. This suggests that energy metabolism in sweet-type melons may be more inclined toward the latter stages of the TCA cycle. Furthermore, the significant elevation of UMP, CDP-glucose, and GMP in the ST group reflected that nucleotide metabolism and sugar activation may be more active. CDP-glucose is a key sugar donor for glycosylation reactions, and its accumulation may enhance the potential for synthesizing glycosylated flavor compounds [32]. This helps explain the enrichment of specific glycosylated substances in the ST group. For instance, well-defined oligosaccharides and glycosides such as b-D-Xylopyranosyl-(1->4)-a-L-rhamnopyranosyl-(1->2)-L-arabinose and (R)-1-O-[b-D-Glucopyranosyl-(1->6)-b-D-glucopyranoside]-1,3-octanediol, though not yet mapped to known metabolic pathways due to a lack of annotation, are likely potential products of this active glycosylation metabolism. Their enrichment is associated with the formation of the sweet taste. Concurrently, differences in nitrogen metabolism and redox homeostasis were observed between the two groups. L-Arginine and glutathione were significantly accumulated in the AT group. Arginine serves not only as a substrate for protein synthesis but also as a precursor for polyamine and nitric oxide (NO) synthesis, involved in plant stress responses [33]. A study on melon flavor also noted that arginine contributes to bitterness, and its accumulation may aid in forming complex flavor profiles [34]. The elevated glutathione level represents a cellular adaptation to oxidative stress, and the redox state can regulate organic acid metabolism. Similar regulatory mechanisms have been observed in other crops, for example, in pepper fruit, exogenous glutathione treatment was shown to modulate the conversion of organic acids to sugars [35]. The possibility cannot be ruled out that the high endogenous accumulation of glutathione in acidic-type melons may similarly participate in reshaping their organic acid metabolic network.

Analysis of the OF vs. GF groups suggested that while overall pathway enrichment pointed to differences in primary metabolism, such as arginine biosynthesis, the basis of color differentiation is more likely associated with specific secondary metabolic pathways related to pigment synthesis, conversion, and stabilization. The significant accumulation of phytol, a constituent of chlorophyll, in the GF group may reflect active degradation of chlorophyll or metabolism of its breakdown products [36,37]. Notably, phytol is also a precursor for tocopherol synthesis [38]. Metabolite data further revealed a more pronounced accumulation of the tocopherol oxidation product α-tocopherolquinone in the OF group. This may be related to the specific redox environment accompanying the substantial accumulation of carotenoids in orange-fleshed fruits [37]. Concurrently, the OF group specifically accumulated higher levels of the C30 apocarotenoid β-citraurinene within the carotenoid metabolic pathway, a red pigment also found in citrus peel [39]. This study also found that another, more structurally complex oxygenated carotenoid derivative, (3S,3′S,5R,5′R,6R)-3,6-epoxy-5,6-dihydro-3′,5,8′-trihydroxy-beta, kappa-caroten-6′-one, was present at higher levels in the OF group, providing metabolic clues for the overall activation of carotenoid synthesis and modification pathways in orange-fleshed melon. Similar observations have been made in other cucurbit crops. For instance, orange flesh in pumpkin is associated with the accumulation of carotenoids such as lutein, β-carotene, and zeaxanthin, and orange-fleshed watermelon is characterized by high β-carotene content [40,41]. Furthermore, the OF group was enriched with coumarin and 4-hydroxybenzaldehyde. These substances, together with the oxidized α-tocopherolquinone, point toward a more active antioxidant metabolic environment, which may help stabilize carotenoids [42]. Simultaneously, the accumulation of the arginine biosynthesis intermediates citrulline and ornithine provides a potential nitrogen metabolic and energetic foundation for this vigorous pigment synthesis process [43]. In contrast, the GF group accumulated a greater abundance of terpenoids, including monoterpenes such as trans-allo-ocimene and α-terpineol propanoate, as well as sesquiterpenes and diterpenes like costunolide, germacrenone, and cafestol. These compounds often possess volatile aroma properties or known plant defense functions [44,45]. From a biosynthetic perspective, carotenoids and these terpenoids share the upstream methylerythritol phosphate (MEP) pathway, competing for common precursors [46,47]. On this basis, it is hypothesized that orange-fleshed melon channels more terpenoid precursors into carotenoid pigment production, whereas green-fleshed melon allocates a greater share toward the synthesis of volatile and defensive terpenoid compounds.

Overall, sensory evaluation provided a perception-based data foundation for this study. Integrating instrumental analysis technologies such as electronic tongue, electronic nose, and GC-MS for standardized measurement of taste and aroma components, coupled with cross-referencing to sensory scores, will help more stably decipher the chemical basis of flavor composition [48,49]. Indeed, studies on various fruits and vegetables have successfully combined subjective and objective evaluation methods, serving as a valuable reference for subsequent in-depth exploration [50,51]. Furthermore, while metabolomic analysis is powerful in revealing metabolite differences, its interpretation is currently constrained by the coverage of existing databases. Even for well-studied model organisms, a considerable proportion of detected signals cannot be definitively identified in current databases [52]. For plant species like melon with rich secondary metabolism, the coverage of public databases is even less comprehensive. This has led to difficulties in annotating some identified differential metabolites to standard KEGG pathways, thereby limiting both the systematic interpretation of the associated metabolic networks and the in-depth exploration of their functional components [53]. This highlights the inherent tension between the strength of untargeted metabolomics in discovering novel substances and the current limitation in their subsequent biological interpretation. Therefore, the mention and discussion of some unannotated metabolites in this study serve not only to report the boundaries of the current analysis but, more importantly, to provide crucial data and a comparative foundation for future research (Table S9). Given the relatively small size of the sensory panel in this study, training and consistency verification were carried out to ensure data validity to the extent possible; nonetheless, future studies would benefit from larger panels combined with instrumental analysis. In addition, due to the limited sample size, future research could expand the number of samples and perform targeted quantitative analysis of the key differential metabolites identified here, such as phytol, α-tocopherolquinone, and β-citraurinene. Integrating multi-omics approaches to elucidate their regulatory networks would provide a precise metabolomic foundation for melon quality assessment, taste/flavor modulation, and product development. As plant metabolite databases continue to expand, annotation standards become unified, and cross-species comparative metabolomics advances, the understanding of the metabolic basis underlying melon flavor and color will undoubtedly become more comprehensive and profound [54].

## 5. Conclusions

This study provided initial insights into the complex metabolic basis underlying melon taste and color formation through sensory-guided metabolomic analysis. The results suggest that scientific grouping based on sensory phenotypes can effectively identify metabolite differences associated with key consumer traits. In terms of taste, the TCA cycle was identified as a key pathway distinguishing sweet and acidic taste profiles. The marked accumulation of citric acid in the AT group may contribute to its perceived sourness, and the coordinated accumulation of citric acid and cis-aconitic acid suggests a possible metabolic bottleneck at the conversion step from citric acid to isocitrate. In contrast, the ST group was characterized by the accumulation of downstream intermediates such as succinic acid and fumaric acid. Regarding color, the divergence between orange and green flesh phenotypes was mainly reflected in the balance between carotenoid synthesis and chlorophyll degradation. The activation of the carotenoid synthesis pathway and a stable antioxidant environment may underlie color development in orange-fleshed melon. In contrast, green-fleshed melon exhibited accumulation of the chlorophyll degradation product phytol and relative enrichment of volatile terpenoids. This suggests that the differential allocation of shared precursors into distinct secondary metabolic branches may be associated with color differentiation. In summary, this study established a research pathway connecting sensory experience to metabolic mechanisms and revealed the key metabolic differences closely associated with core melon quality traits. These findings enhance the metabolomic-level understanding of the mechanisms governing taste and color formation in melon.

## Data Availability

The original contributions presented in this study are included in the article/Supplementary Materials; further inquiries can be directed to the corresponding authors.

## Supplementary Materials

The following supporting information can be downloaded at: https://www.mdpi.com/article/10.3390/metabo16060368/s1, Figure S1: Cross-sections of twelve melon cultivars; Figure S2: Permutation test validation plots of OPLS-DA models for ST vs. AT (a) and OF vs. GF (b) comparisons (*n* = 200); Figure S3: ROC curves of OPLS-DA models for ST vs. AT (a) and OF vs. GF (b); Table S1: Sensory evaluation scores; Table S2: Summary of metabolite annotation, classification, and peak areas; Table S3: OPLS-DA model validation results for ST vs. AT; Table S4: OPLS-DA model validation results for OF vs. GF; Table S5: DAMs in the ST vs. AT comparison; Table S6: DAMs in the OF vs. GF comparison; Table S7: KEGG enrichment analysis results of DAMs in the ST vs. AT comparison; Table S8: KEGG enrichment analysis results of DAMs in the OF vs. GF comparison; Table S9: Unannotated metabolic features with associated peak intensities.
