# Supplementary figures and images for: Integrating Sensory Evaluation and Metabolomics to Reveal the Metabolic Basis of Taste and Flesh Color in Melon (Cucumis melo L.)
Source: Metabolites. 2026 May 28;16(6):368. doi: 10.3390/metabo16060368 (PMC13302874; doi:10.3390/metabo16060368)

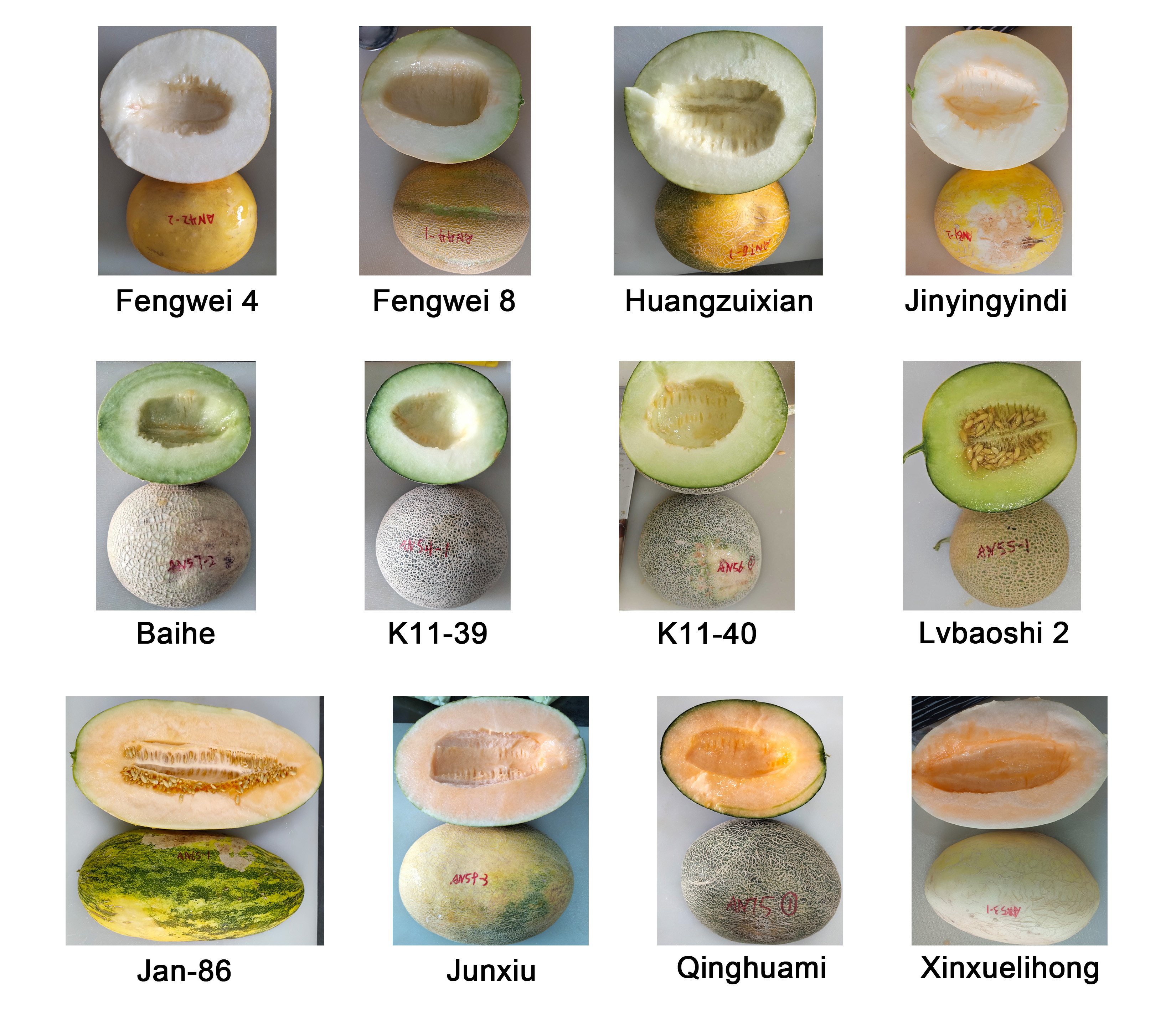

Supplement: Supplementary file 1 [file metabolites-16-00368-s001.zip › Figure S1. Cross-sections of twelve melon cultivars.jpg]

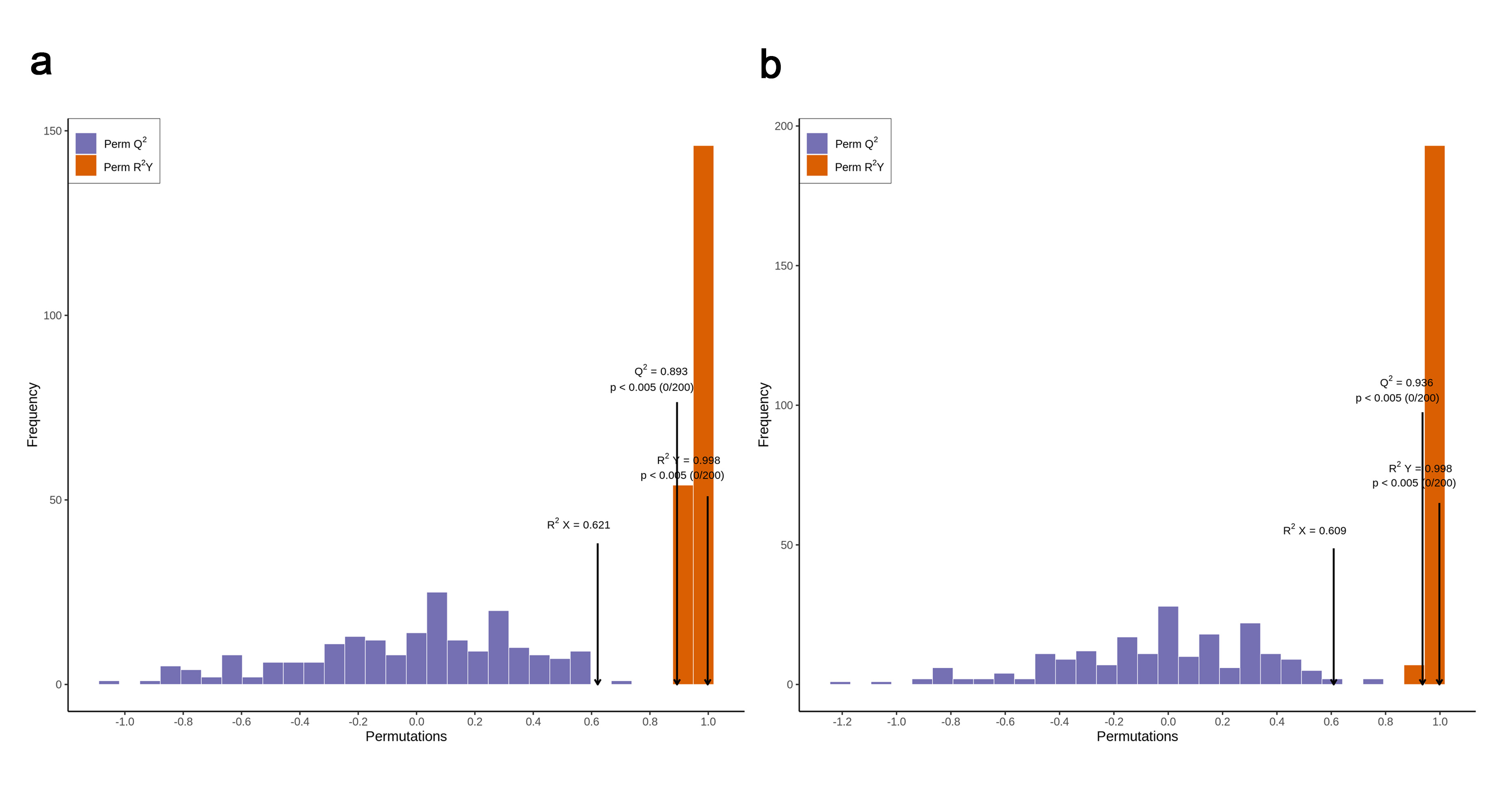

Supplement: Supplementary file 1 [file metabolites-16-00368-s001.zip › Figure S2. Permutation test validation plots of OPLS-DA models for ST vs. AT (a) and OF vs. GF (b) comparisons (n = 200).jpg]

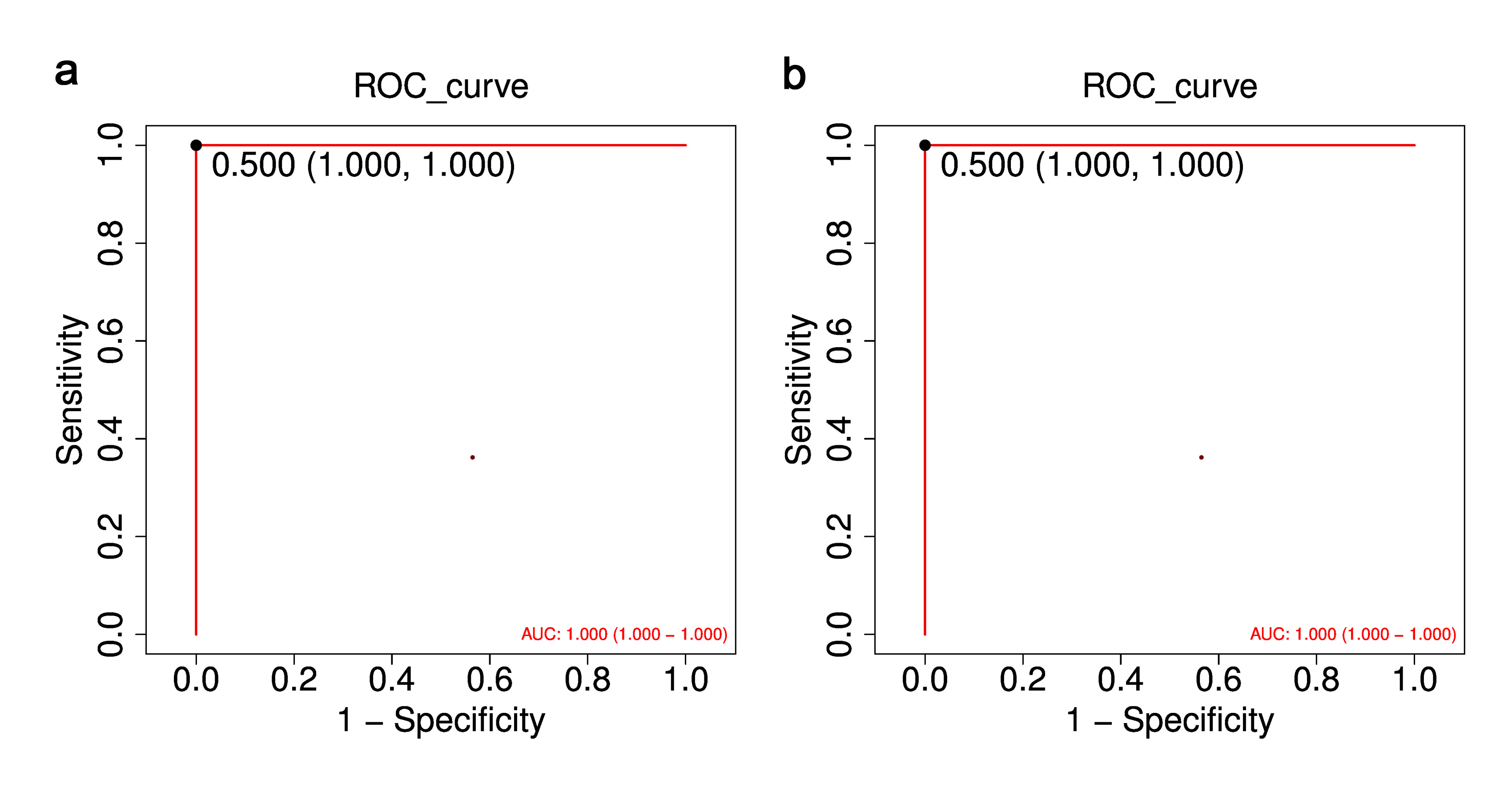

Supplement: Supplementary file 1 [file metabolites-16-00368-s001.zip › Figure S3. ROC curves of OPLS-DA models for ST vs. AT (a) and OF vs. GF (b).jpg]
